# Large bladder flap haematoma following a caesarean section associated with right hydroureteronephrosis: A case report and a mini‑review of the literature

**DOI:** 10.3892/mi.2023.108

**Published:** 2023-09-05

**Authors:** Efthymia Thanasa, Anna Thanasa, Evangelos Kamaretsos, Ioannis Paraoulakis, Vasiliki Grapsidi, Evangelos-Ektoras Gerokostas, Ioannis Thanasas

**Affiliations:** 1 Department of Health Sciences, Medical School, Aristotle University of Thessaloniki, 54124 Thessaloniki, Greece; 2 Department of Obstetrics and Gynecology, General Hospital of Trikala, 42100 Trikala, Greece

**Keywords:** post-caesarean bladder flap haematoma, vesicouterine pouch, caesarean section, imaging, medical treatment, re-laparotomy

## Abstract

Post-caesarean section bladder flap haematoma is a rare postpartum complication. There are currently no specific treatment protocols, at least to the best of our knowledge. In general, the failure of conservative treatment with antibiotics requires the re-operation and surgical drainage of the haematoma. The present study describes the case of a primiparous pregnant woman who, at 40 weeks of pregnancy, delivered by caesarean section. On the 3rd post-operative day, the puerperant, haemodynamically stable, developed febrile infection. During the evaluation, the presence of bladder flap haematoma associated with moderate right hydroureteronephrosis was found. The failure of conservative management led to the decision to perform a re-laparotomy 1 week later. During the surgery, a large bladder flap haematoma was found with a retroperitoneal extension into the right parametrium. The surgical drainage of the haematoma and thorough haemostasis in the area of the vesicouterine pouch was performed. The patient was discharged from the clinic on the 5th post-operative day following the re-operation. After 2 weeks, an ultrasound revealed the complete repair of the lesions in the vesicouterine pouch and the right kidney. In the present study, a brief review of literature is also provided regarding the diagnostic and therapeutic management of patients with post-caesarean section bladder flap hematoma.

## Introduction

Caesarean section, whose prevalence worldwide has increased significantly in recent years, particularly when medically indicated, is a life-saving procedure. In numerous cases, however, it can lead to unexpected short-term or long-term complications of varying frequency and severity, and can significantly increase maternal and neonatal morbidity and mortality (1). A rare complication of low transverse caesarean section is bladder flap hematoma, which is the result of bleeding of the uterine closure. The localization of the haematoma is usually between the bladder and the lower uterine segment. On more rare occasions, the haematoma may extend under the uterine serosa along the broad ligaments to the retroperitoneal space. The formation of bladder flap haematoma is more frequent during the conventional method of caesarean section, which is characterised by the closure of the visceral peritoneum compared to the Misgav Ladach or Stark method, in which no suturing of the vesicouterine fold to the uterine serosa is required (2).

The present study describes a case which emphasises the significant challenges in the management of patients with post-caesarean bladder flap haematoma. At the same time, it is highlighted that, despite its rarity, the association of hydroureteronephrosis and post-caesarean bladder flap hematoma without closure of the visceral peritoneum is necessary to be included in the differential diagnosis of a puerperant presenting signs of intra-abdominal infection in the immediate post-operative period, even when this is not accompanied by hemodynamic instability. Furthermore, it is underlined that early diagnosis and appropriate treatment can reduce maternal morbidity and mortality significantly.

## Case report

A primiparous pregnant woman, 19 years of age, with inadequate prenatal care and a free personal medical history, at the 40th gestational week, underwent an emergency caesarean section. The patient visited the Gynaecology Outpatient Clinic of the Hospital of Trikala for the first time and had inadequate prenatal care. She did not provide any previous tests that may have been performed during the pregnancy. She also reported that she had not undergone either a first trimester ultrasound nor 2nd level ultrasound. She had not performed a doppler or specific blood tests as a glucose challenge test. A foetal biometry during the ultrasound performed at the Gynaecology-Obstetrics Outpatient Clinic of the Hospital of Trikala revealed that the estimated foetal weight (EFW) was below the 10th percentile and also revealed that the patient had oligohydramnios. The cardiotocography was non-reassuring and revealed a pattern of chronic hypoxia. The aforementioned findings were suggestive of possible foetal growth restriction. The pregnant woman was not in labour and she did not have ruptured membranes. A vaginal culture collected from the patient was negative for group B *Streptococcus*. The pregnant woman had no fever pre-operatively. Indications for performing the caesarean section were signs of chronic foetal hypoxia (oligohydramnios, non-reassuring cardiotocography pattern) combined with the low Bishop Score of the pregnant woman and the assessed foetal growth restriction. Intraoperatively, after suturing the Kerr incision of the uterus and following the careful haemostasis in the area of the vesicouterine pouch, the vesicouterine fold was not re-sutured. The estimated intraoperative blood loss from the caesarean section was ~500 cc. A healthy male neonate with a body weight of 2,450 g was successfully born. There was no puncture or incision of the uterine artery during the caesarean section. After suturing the uterine incision and before the abdominal wall was closed, a careful overview was made for any bleeding from the Kerr incision, and no active bleeding was found. On post-operative day 3, the puerperant developed fever up to 38.2˚C and atypical abdominal pain. Characteristically, continuous severe pain was reported by the patient, localized mainly in the right renal region. Fever and pain were accompanied by a marked increase in the levels of inflammatory markers (white blood cell count, 17.2x10^3^/ml; neutrophil value, 76.4%; C-reactive protein, 23 mg/dl; erythrocyte sedimentation rate, 95 mm/h). The haemodynamic status of the puerperant was stable (haematocrit, 35.6%; haemoglobin, 11.7 mg/dl). The results of coagulation tests, urinalysis and renal function tests were within normal range. A blood culture was performed as part of the fever evaluation, which yielded negative results.

**Figure 1:**
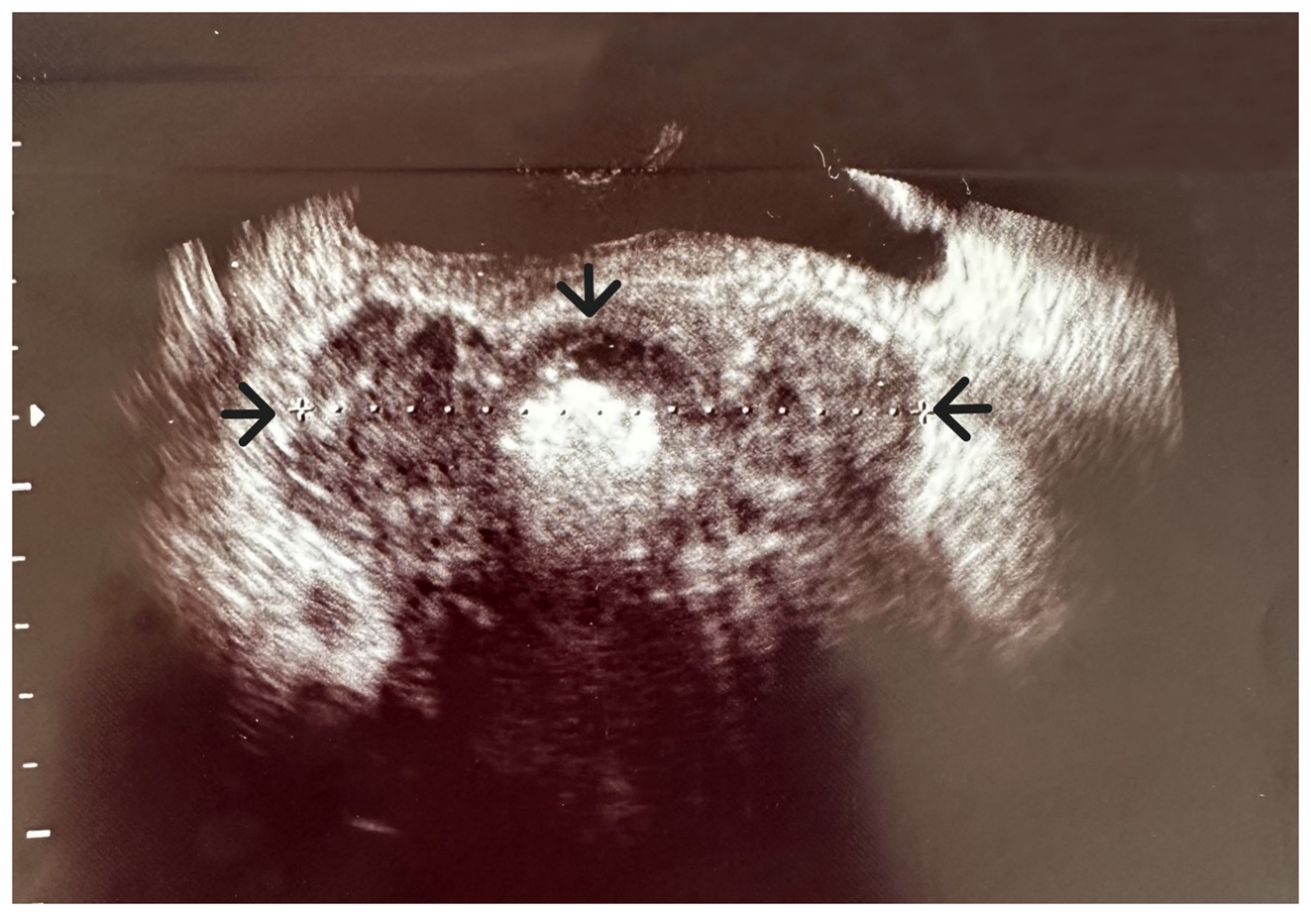
Transvaginal ultrasound image illustrating the bladder flap haematoma (black arrows) of the patient.

**Figure 2:**
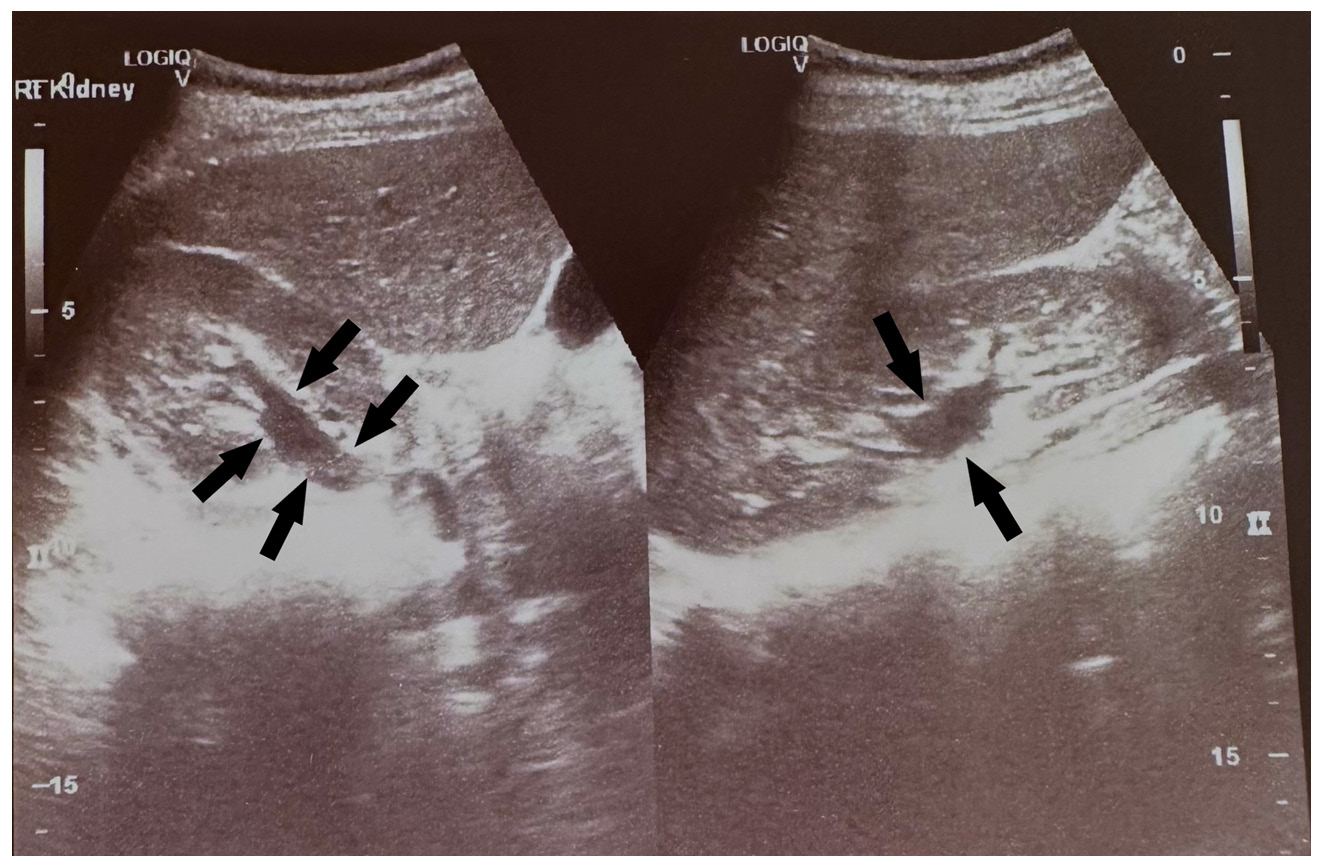
Transabdominal ultrasound image illustrating the dilatation of the right pelvicalyceal system and the proximal ureter (black arrows).

**Figure 3:**
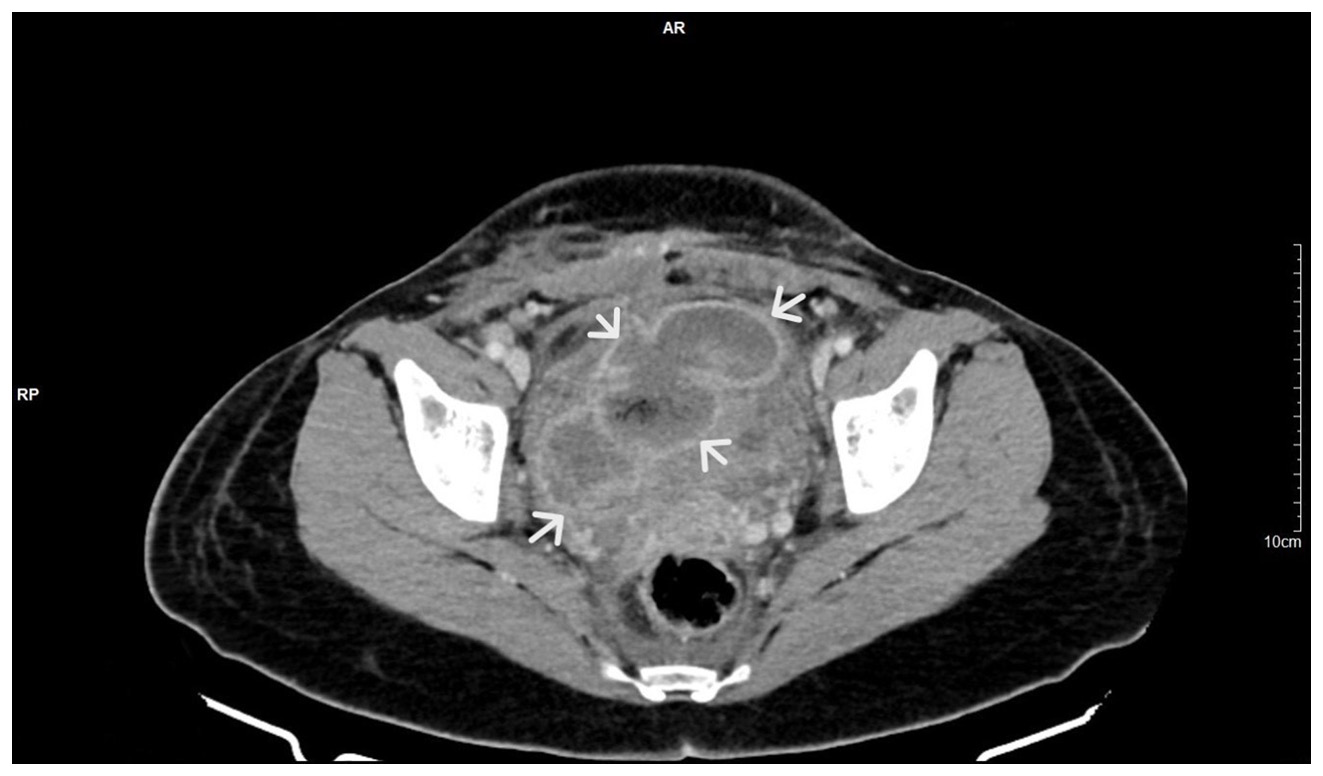
Computed tomography image illustrating a bladder flap haematoma with retroperitoneal extension in the right parametrium (white arrows).

**Figure 4:**
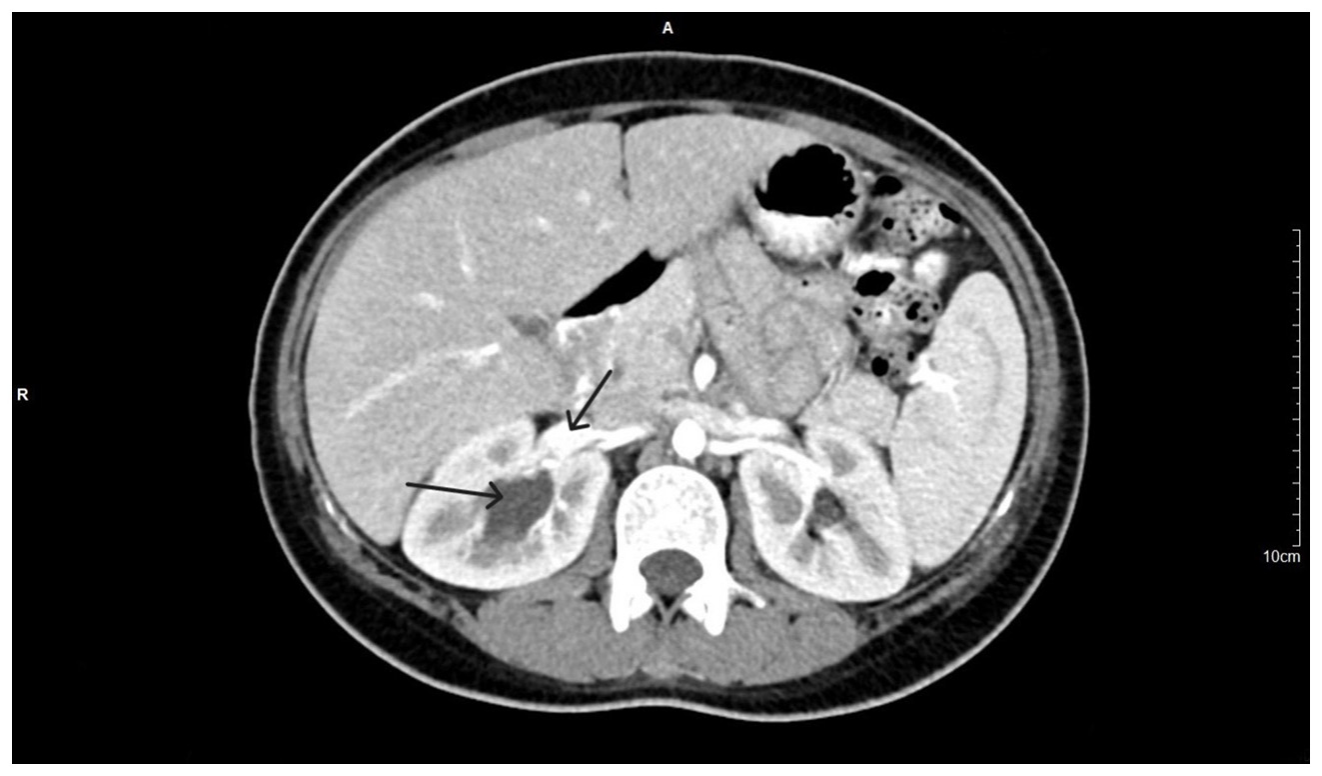
Computed tomography image illustrating the dilatation of the right pelvicalyceal system and the ipsilateral proximal ureter (black arrows) without the presence of urolithiasis.

During the diagnostic evaluation of possible intra-abdominal infection by transvaginal ultrasound, extensive fluid collection with solid components in the vesicouterine pouch along the uterine incision was found (Fig. 1). In addition, ultrasound of the urinary tract revealed moderate pelvicalyceal system dilatation on the right kidney and the ipsilateral proximal ureter without the presence of urolithiasis (Fig. 2). A previous renal ultrasound performed at 35th gestational week and provided by the patient did not reveal any abnormal imaging findings. The computed tomography scan confirmed the findings of the ultrasound. In the area of the vesicouterine pouch, an encapsulated fluid collection was found resembling a haematoma, with dimensions of 110x55 mm and with extensive retroperitoneal extension in the right parametrium (Fig. 3). In addition, the typical dilatation of the right pelvicalyceal system and the ipsilateral proximal ureter was observed (Fig. 4). No arteriogram or venogram was performed as this diagnostic test was not available at the authors' hospital.

For the treatment of the intra-abdominal infection, it was initially decided to intravenously administer a double antibiotic treatment of tigecycline 100 mg/12 h (this antibiotic is effective in complicated intra-abdominal infections) (3) and tazobactam-piperacillin 4 g/8 h, as a broad-spectrum antibiotic. However, as there was no improvement in the patient's clinical condition and taking into consideration the high levels of inflammatory markers of revealed by the blood test results, it was decided to perform a laparotomy on 10th post-operative day following the caesarean section. Intraoperatively, a large bladder flap hematoma was found with retroperitoneal extension to the right parametrium. There were no macroscopic findings consistent with an abscess. A sample of the hematoma was collected for a culture test, which was negative. Drainage of the haematoma was performed, washing away the area with normal saline (0.9%,). A careful overview of the integrity of the incision on the uterine wall and thorough haemostasis were also made. Suction drainages were placed in the vesicouterine pouch and in the pouch of Douglas.

**Figure 5:**
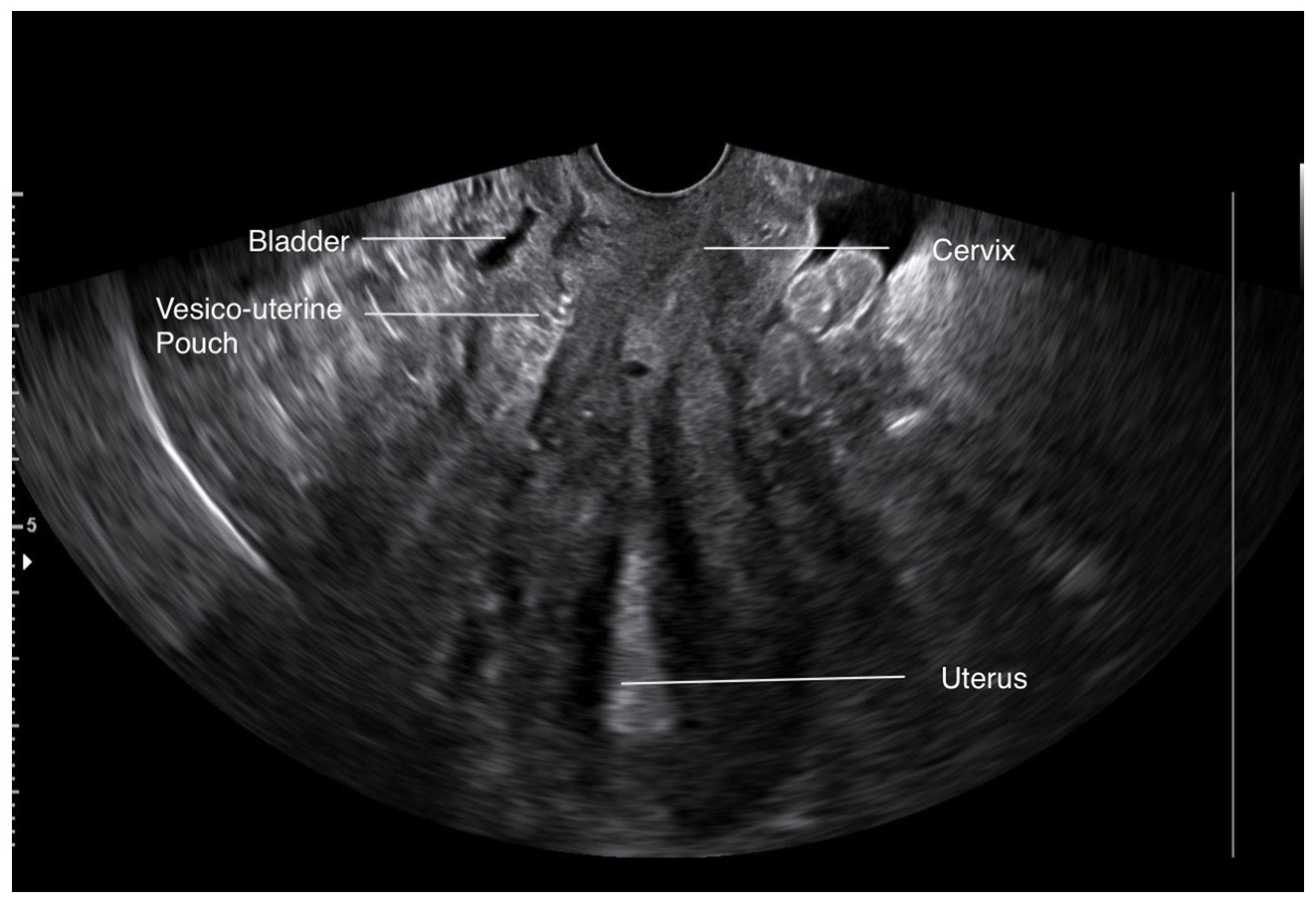
Post-operative transvaginal ultrasound image illustrating the vesicouterine pouch, which reveals the complete repair of the pre-operative lesion.

**Figure 6:**
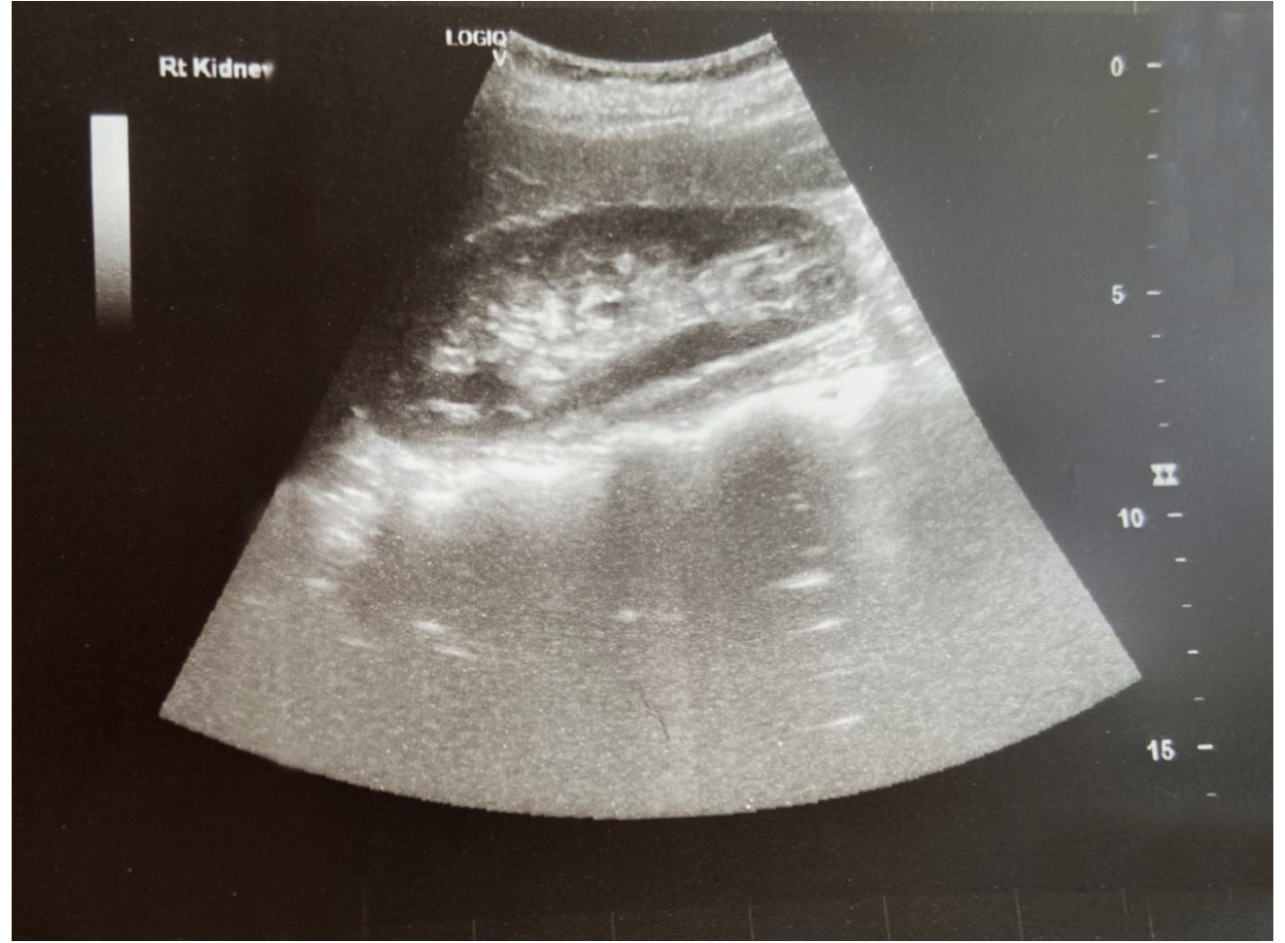
Post-operative ultrasound image of the right kidney, which confirms the absence of hydroureteronephrosis.

During the immediate post-operative period, remission of the fever and a gradual improvement in the levels of inflammation markers were observed (white blood cell count, 8x10^3^/ml; neutrophil value, 68.2%; C-reactive protein, 3.1 mg/dl; erythrocyte sedimentation rate, 15 mm/h). The patient was discharged from the clinic on the 5th post-operative day following the re-operation, and underwent a consultation for re-examination at the gynaecology Outpatient Clinic of Trikala. At 20 days after the re-operation, an ultrasound of the patient revealed that the bladder flap hematoma had been completely absorbed (Fig. 5). The complete repair of the dilatation of the right pelvicalyceal system and the ipsilateral ureter was also observed (Fig. 6). Τhe patient was provided with a thorough consultation about the difficulties that may arise during the next caesarean section and the fact that a general surgeon or a gynaecologic oncologist may be required.

## Discussion

The clinical diagnosis of post-caesarean section bladder flap haematoma is relatively difficult. The haemodynamic instability of the puerperant with signs of hypovolemia (decrease in haemoglobin levels, tachycardia, oliguria) and/or signs of infection (pelvic pain, fever, leukocytosis) could be clinical conditions and laboratory findings that support the formation of bladder flap hematoma, but they are non-specific (4). Similarly, in the patient described herein, despite the formation of a large bladder flap hematoma, the hemodynamic status was stable. The decrease in haemoglobin levels between the initial pre-operative blood tests and those of the third post-operative day was 1.4 mg/dl. In addition, it was uncommon that the formation of an encapsulated hematoma in the vesicouterine pouch occurred after performing a caesarean section without the closure of the visceral peritoneum. In similar cases, the blood loss leaks into the peritoneal cavity, resulting in the formation of a haemoperitoneum, rather than the formation of a bladder flap haematoma. Furthermore, notable is the fact that the lateral extension of the haematoma into the retroperitoneal space of the right parametrium caused the extrinsic compression of the right ureter, resulting in ipsilateral hydroureteronephrosis. To date, at least to the best of our knowledge, there is no report available in the worldwide literature that associates hydroureteronephrosis with the formation of post-caesarean section bladder flap hematoma.

In contrast to clinical conditions and laboratory findings, the diagnosis of bladder flap haematoma is greatly assisted by imaging. Upon an ultrasound, a non-specific solid mass or a mass with solid and cystic components, the size of which varies and may exceed 10 cm (as in the case in the present study), is usually detected between the posterior bladder wall and the anterior wall of the lower uterine segment. However, in any case, the differentiation between a haematoma and an infected hematoma or abscess may be difficult (5). Computed tomography is the method of choice for the imaging of severe post-operative intra-abdominal complications following caesarean section, such as intra-abdominal haematomas, abscesses, bladder flap haematomas, uterine dehiscence-rupture and pelvic thrombophlebitis (6). The role of magnetic resonance imaging is limited to those cases where it is necessary to clarify ultrasound and computed tomography findings, or when there is a strong contraindication to the use of contrast media (7). However, regardless of the imaging method used, it is considered that knowledge of the normal post-operative pelvic anatomy is essential to assist in the optimal timely diagnosis and appropriate management of the complication, in order to avoid unnecessary surgical interventions and adjunctive treatments (8).

To the best of our knowledge, there is no specific, established treatment protocol for the management of bladder flap haematoma available in the current literature. Conservative management with administration of broad-spectrum antibiotics may be the first treatment choice, particularly in patients who are haemodynamically stable. A re-laparotomy to drain the haematoma may be used in those cases where blood loss is severe or when the formation of the haematoma is accompanied by clinical signs of intra-abdominal infection that do not respond to appropriate antibiotic treatment (2). In a previous study, the rate of re-laparotomy following caesarean section was estimated at 1.04%, with a high maternal mortality rate that reached 11.4% (9). The main indication for re-operation was intra-abdominal bleeding (41.7%). This was followed by haematomas and intra-abdominal infections with rates of 29.2 and 7.7%, respectively. Re-laparotomy for the treatment of bladder flap haematoma was not reported (9). In a recent study, active bleeding was the cause of ~78% of re-operations in puerperants who underwent caesarean section. Placenta previa and an emergency caesarean section, particularly when performed during the second stage of labour, have been found to be the main risk factors for re-laparotomy following caesarean section (10). In the present study, as the patient had no risk factors for haemorrhage, the bladder flap hematoma was treated by laparotomy due to the non-responsiveness of the patient to antibiotic treatment (persistent fever and continued increase in inflammation markers). Laparoscopy was not available at the authors' hospital.

Laparoscopic surgery in well-organised and experienced centres is currently an excellent minimally invasive and safe treatment option for the drainage of the bladder flap haematoma and the management of other pelvic haematomas and abscesses. It is considered that laparoscopy for the management of bladder flap haematoma is easier and has higher success rates in those cases where no visceral peritoneal closure has been performed (2). In addition, percutaneous aspiration or surgical transvaginal evacuation may be an alternative conservative treatment option for the management of small pelvic haematomas. Hysterectomy may be considered in cases of sepsis with severe uterine necrosis and irreversible endometritis with formation of extensive multiple abscesses (4,11).

Finally, it is useful to emphasise that despite the widespread use of caesarean section in recent decades, there is no consensus available to date on the optimal surgical technique to be used to minimise complications of the operation, at least to the best of our knowledge. The intraoperative thorough control of haemostasis and post-operative universal antibiotic administration constitute the basis of prophylactic measures to prevent post-operative haemorrhagic complications following caesarean section (4). In addition, the non-closure of the visceral peritoneum after suturing the uterine incision allowing the vesicouterine pouch to communicate with the peritoneal cavity may be a recommendation to avoid formation of bladder flap hematoma (12). Furthermore, eschewing superficial incising and dissecting the peritoneal lining to separate the urinary bladder from the lower uterine segment before uterine incision, which results in the avoidance of bladder flap formation, may currently be a weak recommendation to prevent bladder flap haematoma and reduce maternal morbidity after performing a caesarean section (12).

In conclusion, post-caesarean section bladder flap haematoma without suturing the visceral peritoneum is a rare postpartum complication. The extensive lateral extension of the haematoma into the retroperitoneal space of the parametrium and its association with hydroureteronephrosis due to extrinsic compression of the distal ureter is a unique case report in the international literature. Despite its rarity, the bladder flap hematoma should be included in the differential diagnosis of puerperant presenting signs of intra-abdominal infection, with or without hemodynamic instability, in the immediate post-operative period following a caesarean section, with the aim of reducing maternal morbidity and mortality.

## Data Availability

The data used in the current study are available from the corresponding author upon reasonable request.
